# A *Plasmodium yoelii *soluble factor inhibits the phenotypic maturation of dendritic cells

**DOI:** 10.1186/1475-2875-7-254

**Published:** 2008-12-15

**Authors:** Jamie M Orengo, Kurt A Wong, Carlos Ocaña-Morgner, Ana Rodriguez

**Affiliations:** 1 New York University School of Medicine, Department of Medical Parasitology, 341E. 25th St. New York, NY 10010, USA; 2 Regeneron Pharmaceuticals, Inc. 777 Old Saw Mill River Road, Tarrytown, NY 10591, USA; 3 Institute of Physiological Chemistry, Medical School, MTZ, Dresden University of Technology, Fiedlerstr. 42, 01307 Dresden, Germany

## Abstract

**Background:**

Infection with the protozoan parasite *Plasmodium *is the cause of malaria. *Plasmodium *infects host erythrocytes causing the pathology of the disease. *Plasmodium*-infected erythrocytes can modulate the maturation of dendritic cells (DCs) and alter their capacity to activate T cells.

**Methods:**

Mice infected with *Plasmodium yoelii *and isolated *P. yoelii*-infected erythrocytes were used to study their effect on the maturation of mouse dendritic cells.

**Results:**

DCs are not able to mature in response to LPS injection during the late stage of *P. yoelii *infection in mice, indicating impaired functionality of these cells *in vivo*. *P. yoelii- *infected erythrocytes inhibit the maturation of DCs *in vitro *in a dose-dependent manner, which is consistent with the inhibition found during late infection when parasite burden is highest. The inhibition of DC maturation and the cytokine secretion profile of DCs are modulated by soluble factors released by *P. yoelii*-infected erythrocytes. A small, heat-stable, non-hydrophobic molecule of *P. yoelii*-infected erythrocytes rapidly inhibits the LPS induced phenotypic maturation of DCs in a reversible manner.

**Conclusion:**

These findings add evidence to the malaria associated immune suppression *in vivo *and *in vitro *and provide insight into the nature and mechanism of the *Plasmodium *factor(s) responsible for altering DC functions.

## Background

The interactions of the *Plasmodium *parasite with the immune system of the host are complex in many aspects regarding the activation and regulation of different types of immune cells. The specific characteristics of the immune response may contribute to the successful persistence of the parasite and the slow generation of immunity against the disease that is observed in endemic areas [1]. Inhibition of specific T cell responses to malaria antigens and T cell depletion have been identified in individuals infected with malaria [2-5]. Immune suppression does not appear to affect only anti-*Plasmodium *responses, but the parasite can also inhibit immune responses to other organisms. In fact, malaria is associated with a higher incidence of other infectious diseases [6-8] and reduced immune responses to vaccinations [9,10]. Studies using *in vitro P. falciparum *cultures or *in vivo *murine models with different *Plasmodium *strains have shown that infection is associated with altered macrophage [11-15] and dendritic cell (DC) responses [16-25]. However, other studies have found normal DC responses to *Plasmodium *[26-31].

DCs are antigen presenting cells that play a pivotal role in the initiation of immune responses, as they form a bridge between the innate and adaptive immune responses and possess the unique capacity to initiate primary immune responses via the activation of naïve T cells [32]. Pathogens frequently undermine the immune system by modulating the ability of DCs to initiate an immune response [33]. The maturation and function of monocyte-derived human DCs are modulated by *P. falciparum-*infected erythrocytes *in vitro *[16,18,34]. This phenotype is also supported with field evidence since peripheral blood DCs from children with acute *P. falciparum *infection showed low expression levels of human leukocyte antigen (HLA)-DR on their surface [35,36], suggesting the functional impairment of these DCs. Given the crucial role of DCs in both the innate and adaptive immune responses, modulation of DC functions may provide some explanation as to why protective immunity to malaria is slow to develop.

Using a murine malaria model, it was tested if parasite infection affects the capacity of DCs to respond to another stimulus *in vivo *and observed that the parasite inhibits the phenotypic maturation of splenic DCs in response to lipopolysaccharide (LPS). The *P. yoelii *inhibition of phenotypic DC maturation was dose dependent and contact independent, which is consistent with previous findings using the human *in vitro *model [34]. The culture medium of *P. yoelii*-infected erythrocytes inhibits LPS-induced maturation of DCs and performed the biochemical characterization of a *P. yoelii *factor(s) responsible for this activity.

## Methods

### Reagents, mice and parasites

All chemical reagents were from Sigma unless otherwise specified. All antibodies for flow cytometry were purchased from BD Biosciences. The parasite used was *P. yoelii *17X NL. Female, 6–8 week old Swiss Webster (SW) or BALB/C mice were purchased from Taconic or the National institutes of Health (NIH). Monosodium urate crystals were prepared as previously described [37].

### Isolation of CD11c^+ ^DC from *P. yoelii*-infected mice

For the initiation of parasite infection, mice were injected intraperatonealy (I.P.) with 10^6 ^infected erythrocytes. To evaluate parasitaemia the number of parasitized erythrocytes from 500 cells in a Giemsa-stained thin blood smear was calculated.

*In vivo *maturation of CD11c^+ ^cells was induced by intravenous injection (I.V.) of 25 μg/mouse of lipopolysaccharide (LPS) from *Salmonella typhimurium *diluted in PBS 24 hours before isolation of CD11c^+ ^splenocytes. Control mice were injected with the same volume of PBS.

Whole spleens were aseptically removed from euthanized mice and splenocytes were obtained by mechanical disruption through a cell strainer. Erythrocytes were lysed by incubation with ammonium chloride/potassium hydrogen carbonate buffer (155 mM NH_4_CL, 1 mM KHCO_3_, 0.1 mM Na_2_EDTA) for five minutes followed by washing with cold DMEM (Mediatech) supplemented with 10% FBS (Invitrogen Life Technologies) and PSG antibiotic mix (100 U/ml penicillin, 100 ug/ml streptomycin and 2 mM L-glutamine; Invitrogen Life Technologies). Cells were kept on ice at all times. CD11c+ splenocytes were obtained by positive selection using anti-CD11c antibodies and MACS magnetic columns (Miltenyi Biotec) according to the manufacturer's instructions. After isolation, cells were washed, stained with the appropriate antibodies, and assayed via flow cytometry for the expression of co-stimulatory molecules.

### Flow cytometry analysis

Prior to staining, DCs were incubated with anti-CD16/CD32 (FCγIII/II receptor; 2.4 G) for 5 minutes on ice to prevent the binding of antibodies to Fc receptors. For analysis, the following antibodies were used: PE anti-CD11c (HL3), FITC anti-CD40 (3/23) and FITC anti-CD86 (GL1). All antibodies were used at a 1:100 dilution in PBS+10% FBS and cells were kept on ice during the staining procedure. Flow cytometric analysis was performed using a FACSCalibur (BD Biosciences) and CellQuest (BD Biosciences) or FlowJo (Tree Star) software.

### Isolation of erythrocytes from Plasmodium-infected mice

Whole blood was isolated from infected or uninfected anesthetized SW mice via cardiac puncture and diluted in 100 U heparin/ml PBS. Erythrocytes were washed 3 times with PBS and separated from plasma and white blood cells by centrifugation at 1,800 g for 5 minutes. The buffy coat was removed by pipetting. Erythrocytes were either used as prepared or further processed depending on the experiment.

### Separation of schizonts from *P. yoelii*-infected blood

Washed infected erythrocytes were separated into schizont and non-schizont stages by centrifugation using a 53% Accudenz density gradient solution in PBS (Accurate Chemical and Scientific Corp). Uninfected erythrocytes from a matched mouse were treated in the same way. The purified schizonts or uninfected erythrocytes were centrifuged at 1,800 g for 5 minutes and washed twice with PBS. This procedure results in preparations with higher than 85% schizonts.

### Preparation of the conditioned medium of *P. yoelii*-infected erythrocytes

The conditioned medium was prepared as previously described [38]. Briefly, a culture containing 30% schizonts and 70% of non-schizont stages from a *P. yoelii *infected mouse was incubated in DMEM supplemented with antibiotic mix at a density of 5 × 10^8^/ml for 48 h at 37°C, 5% CO_2_. Cultures were centrifuges at 1,800 g for 5 minutes to collect the supernatant. This supernatant was heated to 100°C for 5 minutes, centrifuged at 1,800 g for 5 minutes and filtered through a 0.2 μm diameter filter. A control conditioned medium was prepared in parallel using erythrocytes from an uninfected mouse. To mimic the conditions of schizont lysis in the infected erythrocytes preparation, a proportion of erythrocytes was separated and lysed by hypo-osmotic treatment with water. Osmolarity was restored and a culture containing 30% lysed uninfected erythrocytes and 70% intact uninfected erythrocytes were incubated in DMEM in the same conditions described for the infected conditioned medium. In all experiments, 10% FBS was added to the conditioned medium prior to its addition to DCs.

### Incubation of DCs with *P. yoelii*-infected erythrocytes or the conditioned medium of *P. yoelii*-infected erythrocytes

Murine myeloid bone marrow-derived DCs were differentiated as previously described [38]. Briefly, bone marrow precursors from femurs and tibias of BALB/c mice were cultured in Petri dishes for 7–10 days at 37°C, 5% CO_2 _in DMEM supplemented with antibiotic mix, 10% FBS, and 30% conditioned medium of the myeloma cell line Ag8653 that expresses recombinant granulocyte monocyte-colony stimulating factor (GMCSF) [39]. This procedure results in preparations with higher than 90% CD11c^+ ^cells, which also present surface CD40, CD80, CD86 and MHC-I and II molecules, which are up-regulated in response to LPS [40].

For experiments involving DC cultured with erythrocytes, DCs (10^6^/ml) were incubated with uninfected or *P. yoelii*-infected erythrocytes at a ratio of 30:1 (iRBC:DC) unless otherwise indicated. Cells were cultured in DMEM supplemented with antibiotic mix, 10% FBS and 5% GM-CSF for 24 hours at 37°C, 5% CO_2_. DCs were then induced (or not) to mature with 100 ng/ml LPS and incubated for an additional 24 hours. Erythrocytes were lysed with ammonium chloride/potassium hydrogen carbonate buffer for 5 minutes. DCs were washed and resuspended in PBS+10% FBS and stained with the appropriate antibodies for analysis of co-stimulatory molecules by flow cytometry.

For experiments involving DC cultured with the conditioned medium of *P. yoelii*-infected erythrocytes, DCs (10^6^/ml) were incubated with the conditioned medium for the indicated amount of time and then incubated with 100 ng/ml LPS for an additional 24 h 37°C, 5% CO_2_. DCs were harvested, washed, resuspended in PBS+10% FBS, and stained with the antibodies for analysis of co-stimulatory molecules using flow cytometry.

Incubation media from DC-conditioned media co-cultures was collected and assayed by ELISA for the secretion of IL-6 or IL-12 p70 (OPTI-EIA kit, BD biosciences) or PGE_2 _(Cayman Chemicals)

### Characterization and molecular identification of *P. yoelii *active molecules

#### Size fractionation of the conditioned media using Centricon^® ^columns

The conditioned media of uninfected- or *P. yoelii*-infected erythrocytes was fractionated using Centricon^® ^(Millipore) filters of various membrane sizes according the manufacturers instructions. Briefly, centrifugal force drives the separation of solvents and solutes through the membrane filters of varying sizes into the filtrate vial. The conditioned medium was spun on the Centricon^® ^column at 1,800 g for 90 minutes. The column flow through was collected and used in assays with DCs.

#### Dialysis of the conditioned medium using Tube-O-Dialyzers

Using Tube-O-Dialyzers (Geno Technology, Inc) with a molecular weight cut off of 1 kDa, molecules that were smaller than 1 kDa were dialyzed out of the conditioned medium of uninfected- or *P. yoelii*-infected erythrocytes that were each previously size fractionated to smaller than 3 kDa. Samples were dialyzed against DMEM (1:100 vol:vol) for 8 hours and the DMEM was changed every 2 hours.

#### Treatment with DNase I

DNase I (EMD Biosciences) was resuspended in DMEM, without serum, at 10 mg/ml. Endotoxin was removed from DNase I using the EndoClean kit (BioVintage) according to the manufacturer's protocol. Then the DNase I was added to the conditioned media (1 μg/ml) and incubated at 37°C for 2 h. The DNase I was deactivated by boiling the CM for 10 minutes.

#### Purification using Sep-Pak^® ^reversed phase columns

The conditioned medium of uninfected- or *P. yoelii*-infected erythrocytes was passed through Sep-Pak^® ^reverse phase columns that retain hydrophobic molecules. The Sep-Pak^® ^columns were pre-rinsed with 10 mls of 100% ethanol followed by 10 mls of 0.1% acetic acid. The conditioned media was passed through the column and the flow through containing non-hydrophobic molecules was dried and reconstituted in DMEM without salts. The conditioned medium containing hydrobic molecules was eluted with 70% ethanol, dried, and reconstituted in DMEM.

### Statistical analysis

All data were analysed using GraphPad Prism software. Significant differences were determined using student's t-tests or one-way ANOVA. Data were considered significant if p < 0.05.

## Results

### *Plasmodium yoelii *infection inhibits the *in vivo *maturation of CD11c^+ ^splenocytes

**Figure 1:**
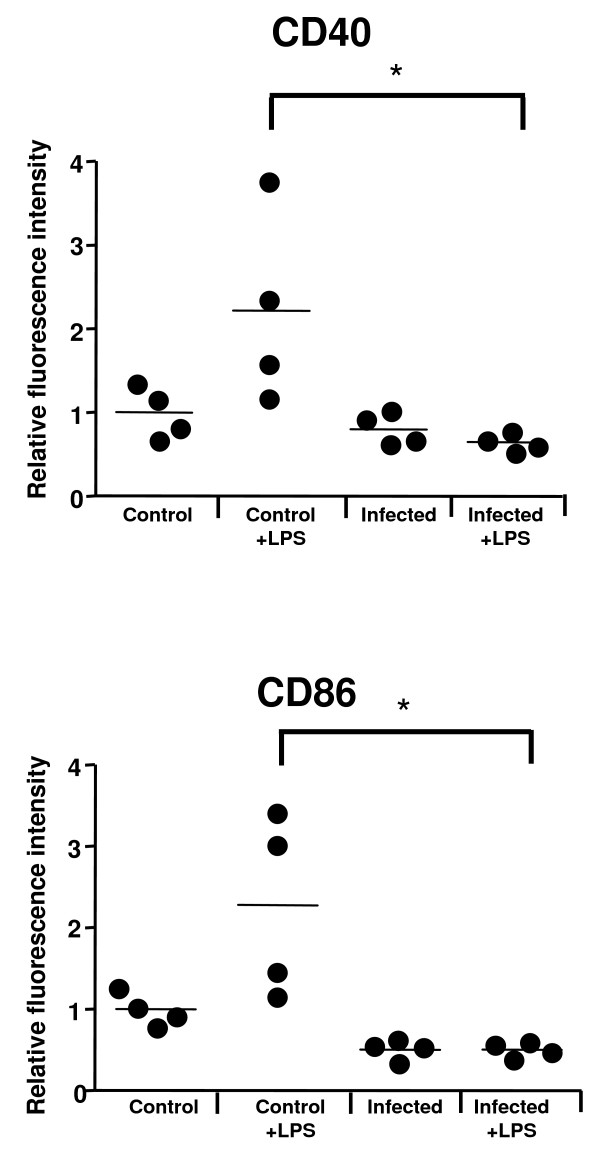
***P. yoelii *infection inhibits the *in vivo *maturation of CD11c^+ ^splenocytes**. SW mice were infected or not with 10^6 ^*P. yoelii*-infected erythrocytes. At day 10 post infection, CD11c^+ ^splenocytes were analysed via flow cytometry for their surface expression of co-stimulatory molecules CD40 and CD86. When indicated, 25 μg/mouse of LPS was injected intravenously 24 h prior to spleen harvest. Results are expressed as relative fluorescent intensity for the gated CD11c^+ ^cells of each mouse compared with the average of gated CD11c^+ ^cells from uninfected, unstimulated mice. Each symbol is representative of one mouse and each group contains at least 3 mice. Bars represent mean value of each group of mice. Differences in surface expression were considered significant when p < 0.05. * represents significant differences in surface expression when compared to LPS treated uninfected mice.

The ability of splenic CD11c^+ ^cells (myeloid DCs) from *P. yoelii-*infected Swiss Webster (SW) mice to respond to a maturation stimulus during late infection *in vivo *was tested. The surface expression of the co-stimulatory molecules CD40 and CD86 was measured as an indication of DC maturation [41]. As reported before for late *P. yoelii *infection [17,19,42], the expression of CD40 and CD86 on CD11c^+ ^splenocytes was not significantly upregulated in response to parasite infection (average parasitaemia, 21.62). When the response to LPS that was injected into the mice 24 h before was analyzed, CD11c^+ ^splenocytes from infected mice did not up-regulate the co-stimulatory molecules CD40 and CD86 (Figure 1). Whereas CD11c^+ ^splenocytes from control uninfected mice increased the expression of these co-stimulatory molecules in response to LPS. These results suggest that infection with *P. yoelii *inhibits CD11c^+ ^responses to other maturation stimuli, such as LPS. It is important to note that during late *P. yoelii *infection the regulatory DC subset is the dominant DC population in the spleen, therefore the data presented here mainly reflect the behavior of this population[42].

### *In vitro *inhibition of DC maturation is dose-dependent

**Figure 2:**
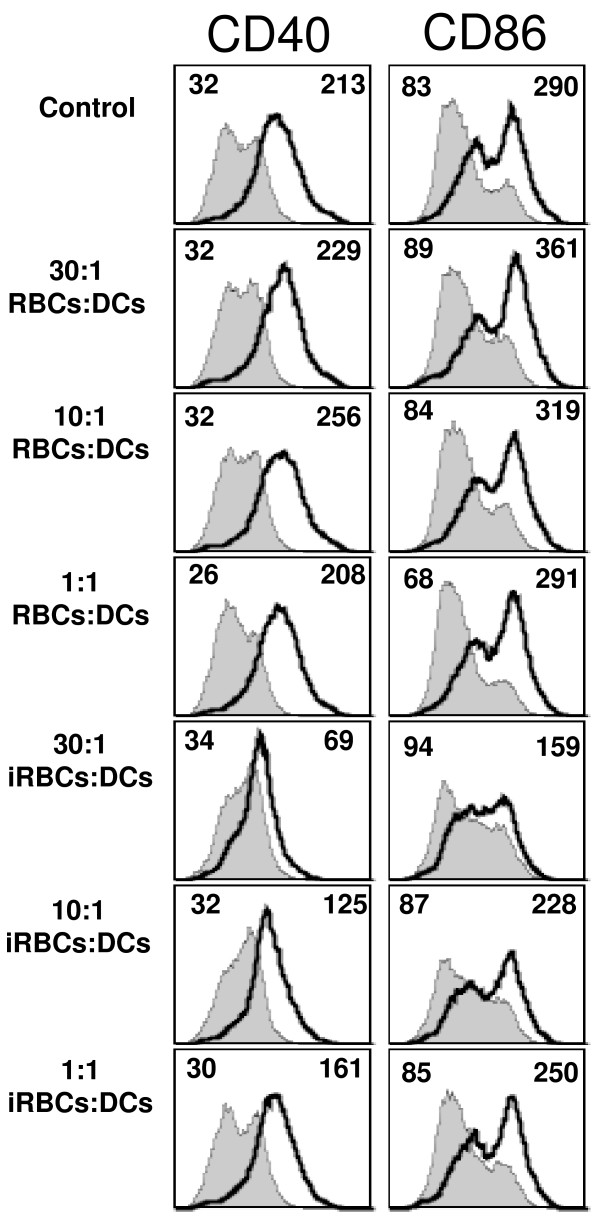
***P. yoelii in vitro *inhibition of DC maturation is dose dependent**. DCs were incubated alone (Control), in the presence of uninfected RBCs (RBCs) or *P. yoelii*-infected erythrocytes (iRBCs) at the indicated ratio to DCs for 24 h followed by an additional 24 h in the presence or absence of 100 ng/ml LPS. FACS plots show CD40 and CD86 surface expression on DCs from control cultures (gray filled histograms) or incubated with LPS (black thick line). Mean fluorescent intensity (MFI) values are indicated in each plot for control and LPS stimulated. Each FACS plot is representative of one of at least 2 independent experiments.

*Plasmodium falciparum *and *P. yoelii-*infected erythrocytes inhibit the LPS induced upregulation of histocompatibility and co-stimulatory molecules on DCs *in vitro *[16,17]. Furthermore, it was shown using the human *in vitro *model that this inhibition was dose dependent [34]. *P. yoelii*-infected erythrocytes, when incubated with murine myeloid DCs at a ratio of 30:1, were found to inhibit the LPS induced upregulation of the co-stimulatory molecules CD40 and CD86 on DC cell surfaces (Figure 2). This failure to up-regulate co-stimulatory molecules was not observed when DCs were incubated alone or with uninfected RBCs at the same ratio. The inhibitory effect was dose-dependent and decreased at lower ratios of *P. yoelii*-infected erythrocytes to DCs (Figure 2). This is consistent with observations using the human *in vitro *model [34]; however, some inhibition of co-stimulatory molecule expression at lower ratios (10:1 and 1:1 iRBC:DCs) that was not apparent in the human experiments was still observed.

### Uric acid and the inhibition of DC maturation by *P. yoelii*-infected erythrocytes

**Figure 3:**
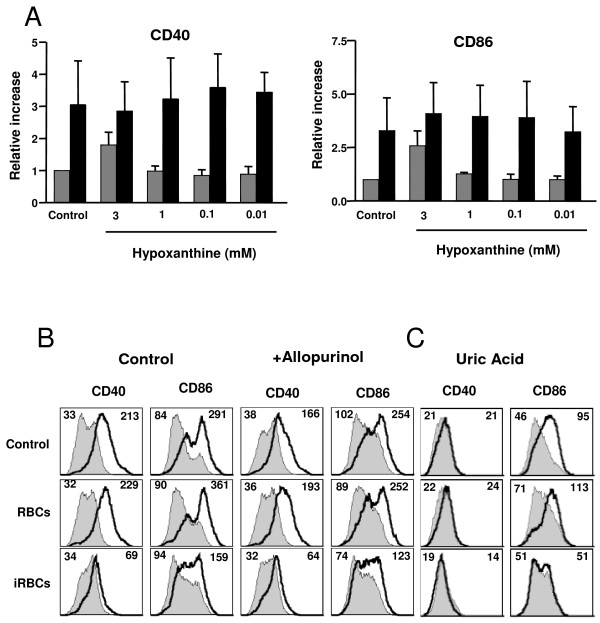
**Uric acid and the inhibition of DC maturation by *P. yoelii-*infected erythrocytes**. (A) DCs were incubated alone (Control) or in the presence of various concentrations of hypoxanthine for 24 h followed by an additional 24 h in the absence (grey bars) or presence (black bars) of 100 ng/ml LPS. Results are expressed as the relative increase in MFI over DCs incubated in media alone. Error bars represent the standard deviation of 3 averaged independent experiments. (B, C) DCs were incubated alone (Control) or with uninfected RBCs (RBCs) or *P. yoelii*-infected erythrocytes (iRBCs). (B) 2 mM allopurinol was added or not to the co-cultures for 24 h followed by an additional 24 h in the presence or absence of 100 ng/ml LPS. (C) Co-cultures were incubated alone for 24 h before addition of uric acid crystals for additional 24 h. FACS plots show CD40 or CD86 surface expression on DCs from control cultures (gray filled histogram) or incubated with LPS (B) or uric acid crystals (C) (black thick line). Mean fluorescent intensity (MFI) values are indicated in each plot for control and stimulated. Each FACS plot is representative of one of at least 2 independent experiments.

DCs produce TNF in response to *Plasmodium *infected erythrocytes [29,30,38]. The *P. yoelii *factor that induces TNF was shown to be *Plasmodium*-derived hypoxanthine that is converted into uric acid by xanthine oxidoreductase present in the serum [38]. Since TNF can induce the maturation of DCs [43] and it was recently shown that systemic TNF in *Plasmodium*-infected mice can impair DC function [25], it was tested whether hypoxanthine could also be responsible for the impaired maturation of DCs. Addition of hypoxanthine to DCs in the presence of serum results in the secretion of TNF from DCs [38], however, it did not inhibit the LPS-induced maturation of DCs (Figure 3A). At high concentrations of hypoxanthine, an increase in surface co-stimulatory molecules was observed. This is expected since the products of hypoxanthine degradation: reactive oxygen species and uric acid can both induce DC maturation [37,44]. Despite this increase in surface markers, DCs were still responsive to LPS-induced maturation (Figure 3A).

Additionally, it was tested whether the hypoxanthine or its degradation products accumulated in the conditioned medium could play a role in the inhibition of DC maturation. Allopurinol is an inhibitor of xanthine oxidoreductase that blocks hypoxanthine degradation and inhibits *P. yoelii*-induced release of TNF by DCs [38]. It was found that allopurinol did not affect *P. yoelii*-induced inhibition of DC maturation (Figure 3B). Therefore, it is unlikely that hypoxanthine or its degradation pathway are responsible for the *P. yoelii*-induced inhibition of DC maturation. On the contrary, it appears that this pathway may contribute to the maturation of DCs. It was then tested whether *P. yoelii*-infected erythrocytes could also inhibit maturation induced by uric acid. Uric acid is a danger signal that in the crystallized form increases the expression of CD86 on the surface of DCs [37]. Uric acid crystals increased the surface expression of CD86, but not of CD40. In the presence of *P. yoelii*-infected erythrocytes, the uric acid-induced upregulation of CD86 was not observed (Figure 3C).

### Conditioned medium from *P. yoelii*-infected erythrocytes inhibits DC maturation

It has been reported using a transwell culture system that *P. falciparum *modulation of DC phenotypic maturation is not contact dependent [34], suggesting that a soluble factor(s) is responsible for the effect.

**Figure 4:**
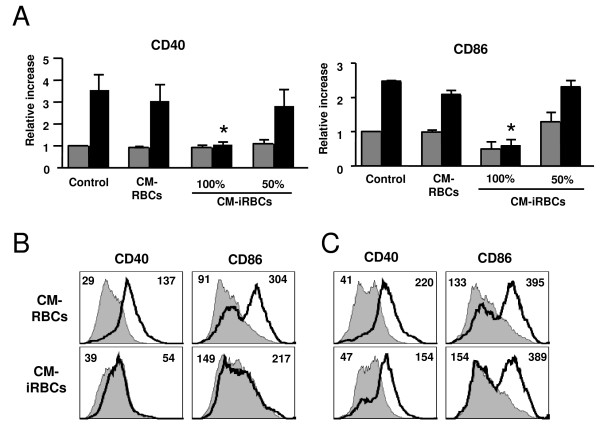
**The conditioned medium of *P. yoelii*-infected erythrocytes inhibits DC maturation**. (A) DCs were incubated alone (Control), in the presence of the conditioned media of uninfected (CM-RBCs) or *P. yoelii*-infected (CM-iRBCs) erythrocytes for 24 h followed by an additional 24 h in the absence (grey bars) or presence (black bars) of 100 ng/ml LPS. The CM-iRBCs was diluted 50% in medium or not (100%). Results are expressed as the relative increase in MFI over DCs incubated in media alone. Error bars represent the standard deviation of 3 averaged independent experiments. *, indicates significant differences in DC surface expression when compared to DC incubated with CM-RBCs and stimulated with LPS. (B) DCs were incubated with either the conditioned medium of uninfected (CM-RBCs) or *P. yoelii*-infected (CM-iRBCs) erythrocytes in the presence or absence of 100 ng/ml LPS for 24 h. (C) DCs were incubated in the presence of the conditioned medium of uninfected (CM-RBCs) or *P. yoelii*-infected (CM-iRBCs) erythrocytes for 24 h. DCs were washed and incubated in media alone for 24 h in the presence or absence of 100 ng/ml LPS. (B, C) FACS plots show CD40 or CD86 surface expression on DCs from control cultures (gray filled histogram) or incubated with LPS (black thick line). Mean fluorescent intensity (MFI) values are indicated in each plot for control and LPS stimulated. Each FACS plot is representative of one of at least 2 independent experiments.

To investigate the possibility that the inhibitory effect observed is also mediated by a soluble factor, the effects of a conditioned medium of *P. yoelii*-infected erythrocytes (described in methods) was tested on DC responses to LPS. The conditioned medium of *P. yoelii*-infected erythrocytes inhibited the LPS induced up-regulation of CD40 and CD86 on the surface of DCs (Figure 4A). A conditioned medium obtained from the incubation of whole uninfected erythrocytes did not inhibit the maturation of DCs. However, as a better experimental control, a proportion of the uninfected erythrocytes was lysed to account for erythrocyte factors that are released as a result of erythrocyte rupture that occurs in the infected erythrocyte preparation. This control conditioned medium did not inhibit the LPS induced upregulation of CD40 or CD86 on the surface of DCs (Figure 4A). The inhibitory effect of the conditioned medium is dose dependent (Figure 4A) and heat stable, since the inhibitory activity was not affected after boiling the conditioned medium before incubation with DCs. These results indicate that a heat-stable soluble factor(s) derived from *P. yoelii*-infected erythrocytes inhibits the maturation of DCs.

To inhibit DC maturation, *P. falciparum *and *P. yoelii*-infected erythrocytes are pre-incubated with DCs for 20 to 24 h prior to the addition of a maturation stimulus [16,17,34]. This suggests that either the factor (s) responsible for inhibiting DC maturation requires time to reach a threshold concentration in the medium or that the DCs require time to respond to it. To prepare the *P. yoelii*-conditioned medium, parasites are incubated in media for 48 h. This is enough time for the inhibitory factor(s) to accumulate in the medium, as evidenced by the inhibitory response obtained (Figure 4A). This experiment, however, was performed by pre-incubating the DCs with the conditioned medium for 24 h before adding the maturation stimulus, as was done with whole infected erythrocytes. To determine the time of action of the soluble factor(s) on DC maturation, the conditioned medium of *P. yoelii *infected erythrocytes was added to DCs at the same time as LPS. In these conditions, a strong inhibition of the LPS-induced maturation was found (Figure 4B), suggesting that the factor(s) has a rapid effect on DCs.

When DCs were pre-incubated with the conditioned medium for 24 h, washed and then incubated with DC medium in the presence or absence of LPS for an additional 24 h, the inhibitory effect of the conditioned medium was not observed, as these DCs upregulated CD40 and CD86 in response to LPS stimulation (Figure 4C). These data suggest that the inhibitory effect of the *P. yoelii *factor(s) on DC maturation is reversible. Since the DCs matured in response to LPS once the conditioned medium was removed, it also suggests that the *P. yoelii-*soluble factor(s) is not toxic to the DCs. It was confirmed that the conditioned medium of *P. yoelii*-infected erythrocytes did not induce DC death. Using propidium iodide exclusion to determine the percentage of live DCs, no differences were found after incubation for 48 h alone or with the conditioned medium from infected or uninfected erythrocytes.

### Conditioned medium modulates DC cytokine production

**Figure 5:**
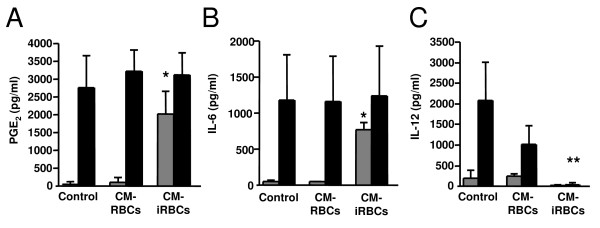
**The conditioned medium of *P. yoelii-*infected erythrocytes modulates DC cytokine production**. (A-C) DCs were incubated with medium alone (control), the conditioned medium of uninfected (CM-RBCs) or *P. yoelii*-infected (CM-iRBCs) erythrocytes for 24 h in the absence (grey bars) or presence (black bars) of 100 ng/ml LPS. (A) PGE_2_, (B) IL-6 or (C) IL-12 p70 concentrations were determined in the incubation medium by ELISA. Error bars represent the standard deviation of 3 averaged independent experiments. *, represents significant differences in IL-6 or PGE_2 _production by DCs in the presence of CM-iRBCs when compared to production in the presence of CM-RBCs. **, represents significant differences in IL-12 production by DCs in the presence of CM-iRBCs with LPS when compared to production in the presence of CM-RBCs with LPS.

The effect of the conditioned medium of *P. yoelii *infected erythrocytes was tested on the secretion of two inflammatory mediators that are released by DCs in response to *P. yoelii*-infected erythrocytes: prostaglandin E_2 _(PGE_2_) [45] and interleukin (IL)-6 [19]. The conditioned medium of *P. yoelii*-infected erythrocytes induced DC production of PGE_2 _(Figure 5A) and IL-6 (Figure 5B), whereas the conditioned medium of uninfected erythrocytes did not induce the production of these inflammatory mediators. The conditioned medium of *P. yoelii*-infected erythrocytes also induces the secretion of TNF from DCs [38].

Pre-incubation of DCs with *P. yoelii*- or *P. falciparum*-infected erythrocytes does not induce IL-12 production, but instead inhibits the secretion of IL-12 in response to LPS [17,18,34]. The incubation of DCs with the conditioned medium of *P. yoelii *infected erythrocytes also resulted in a failure of these cells to secrete IL-12 in response to stimulation with LPS (Figure 5C). Moreover, this was a rapid inhibition, as the conditioned medium was added concurrently with LPS.

### Characterization of the *P. yoelii *soluble factor(s) that inhibits DC maturation

**Figure 6:**
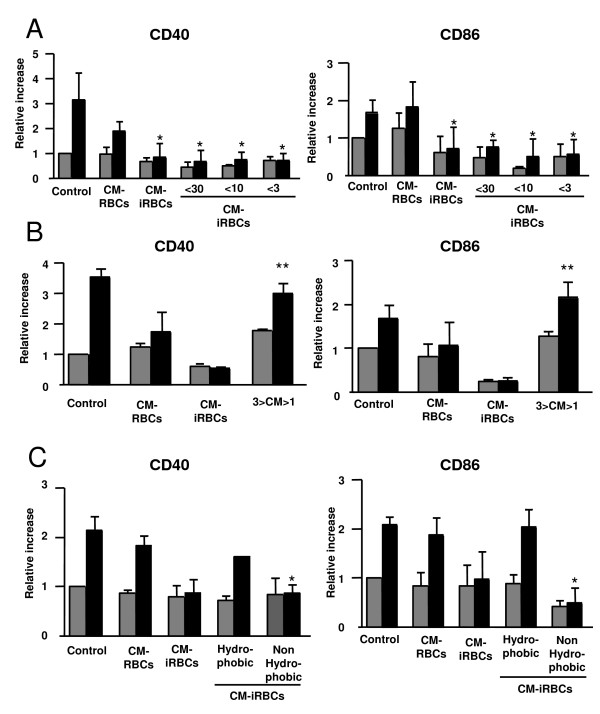
**Characterization of the *P. yoelii *soluble factor that inhibits DC maturation**. (A-C) DCs were incubated alone (Control), in the presence of the conditioned media of uninfected (CM-RBCs) or *P. yoelii*-infected (CM-iRBCs) erythrocytes for 24 h followed by an additional 24 h in the absence (grey bars) or presence (black bars) of 100 ng/ml LPS. (A) Prior to incubation with DCs, conditioned medium of *P. yoelii*-infected erythrocytes (CM-iRBCs) was size fractioned using Centricon^® ^filters to smaller than 30 kDa (<30), 10 kDa (<10) and smaller than 3 kDa (<3). (B) Prior to incubation with DCs, molecules of smaller than 1 kDa were dialyzed out of the *P. yoelii *conditioned medium that was previously size fractioned to smaller than 3 kDa (3>CM>1). (C) Prior to incubation with DCs, The conditioned medium was fractionated based on hydrophobicity using Sep-Pak^® ^columns. (A-C) Results are expressed as the relative increase in MFI over DCs incubated in media alone. Error bars represent the standard deviation of 3 averaged independent experiments. *, represents significant differences in DC surface expression when compared to surface expression on DCs incubated with CM-RBCs and matured with LPS. **, represents significant differences in DC surface expression when compared to surface expression on DCs incubated with CM-iRBCs and matured with LPS.

To further characterize the *P. yoelii *factor(s) that inhibits DC maturation, the conditioned medium was fractionated by size using Centricon^® ^filters of various membrane pore sizes prior to incubation with DCs. When the *P. yoelii*-conditioned medium was size fractionated to smaller than 30 kDa, 10 kDa or 3 kDa there was inhibition of the LPS-induced up-regulation of CD40 and CD86 (Figure 6A). No inhibitory activity was found in the high molecular weight (>30 kDa, 10 kDa or 3 kDa) fractions.

Molecules smaller than 1 kDa were dialyzed out from the *P. yoelii*-conditioned medium that was previously size fractionated to smaller than 3 kDa using Tube-O-Dialyzers^® ^dialysis tubes. When this fractionated, dialyzed conditioned medium was incubated with DCs, the inhibitory effect was lost and the DCs up-regulated surface expression of CD40 and CD86 in response to LPS stimulation (Figure 6B), indicating that the inhibitory molecule(s) was smaller than 1 kDa. The inhibitory activity was also found not to be sensitive to extensive treatment with DNase I.

The non-hydrophobic fraction of the conditioned medium of *P. yoelii*-infected erythrocytes retained the ability to inhibit the LPS induced upregulation of CD40 and CD86 on the surface of DCs (Figure 6C). Conversely, the conditioned medium containing the hydrophobic molecules did not inhibit the LPS induced upregulation of CD40 and CD86 to the DC surface (Figure 6C).

These data suggest that a small, soluble, non-hydrophobic molecule of *P. yoelii *infected erythrocytes inhibits the LPS induced phenotypic maturation of DCs.

## Discussion

A number of studies have analysed the response of DCs incubated with human or mouse *Plasmodium*-infected erythrocytes *in vitro *or obtained from *Plasmodium*-infected mice *ex vivo*. Some of these studies found a normal DC maturation response [26-31,46], but others have found inhibition of DC maturation [16-21,23,25]. This apparent controversy has been resolved recently since it was found that the behavior of DCs is dependent on the strain of infecting parasite [24] and the subpopulation of DC analysed [42]. Additionally, the initial dose of parasite inoculated to induce infection and the time when DCs are analysed during infection influences the maturation state of DCs [42].

In this study, the DC response to a maturation stimulus *in vivo *in infected mice during late *P. yoelii *infection was analised. In these conditions, splenic DCs are found in an immature state and do not respond to LPS injection. It has been shown that DCs become refractory to TLR-mediated secretion of IL-12 and TNF during late *P. yoelii *infection [46]. This study also indicates that DCs are refractory to LPS stimulation. Since the DCs are found in an immature state, it suggests that there is a parasite-induced active inhibition rather than a tolerogenic effect caused by previous *P. yoelii *DC activation.

Recently we showed that *Plasmodium*-derived uric acid induces DC production of TNF [38]. Uric acid crystals have been identified as a danger signal that induces DC maturation [37]. It was found that in the presence of *P. yoelii *infected erythrocytes, DCs do not phenotypically mature in response to uric acid, which further broadens malaria induced suppression implications.

The inhibition of LPS-induced DC maturation was dependent on the dose of *P. yoelii *infected erythrocytes. This is consistent with findings observed using human DCs and *P. falciparum *[34] and suggests that this inhibitory effect would be most relevant during late infection when parasite loads are higher.

Using conditioned medium of *P. yoelii*-infected erythrocytes, it was determined that the *P. yoelii*-infected erythrocyte factor(s) that inhibits the LPS-induced phenotypic maturation of DCs was soluble. Upon further characterization, this factor was found to be heat stable, smaller than 1 kDa and non-hydrophobic. It is possible that this factor is produced by the parasite itself, or alternatively, *Plasmodium *could induce its production by host erythrocytes upon infection. It is not clear whether this factor would perform the same activity during infection *in vivo *since it requires a high concentration of infected erythrocytes to reach activity *in vitro*. However, infected erythrocytes accumulate in certain organs during infection [47], where local concentrations of the inhibitory factor may be reached.

It has been proposed that the inhibition of DC maturation is mediated by the binding of the *P. falciparum *protein PfEMP1, which is expressed on the surface of infected erythrocytes [48], to CD36 on the surface of DCs [16]. This was based on the observation that a non-adherent parasite cell line, which does not express PfEMP1, does not inhibit the maturation of DCs [16]. In addition, the authors observed that antibodies to CD36 inhibited maturation of DCs in a similar manner to *P. falciparum*-infected erythrocytes [35]. Conversely, it has been reported that *P. falciparum *modulation of DC phenotypic maturation is not contact dependent and does not required binding of CD36 and PfEMP1[34], suggesting that a soluble factor(s) is responsible for the effect. Since only a small subset of murine DCs express CD36 [49,50] and *P. yoelii *does not have genes homologous to PfEMP1 [51], the modulation of DCs by a soluble factor(s) derived from *P. yoelii*-infected erythrocytes is likely homologous to the later mechanism [34].

An immune suppressive role for hemozoin has been proposed [52,53], including an inhibitory effect on DC maturation [20,23,54]. However, other reports propose hemozoin as an immune activator because of its ability as a purified extract to enhance DC maturation [55,56]. Recently, it was shown that parasite DNA that is associated with hemozoin after release from ruptured infected erythrocytes is responsible for the activation of DCs [57]. Haemozoin is not likely to be responsible for the DC inhibition observed in this work, since the size fractionation experiments showed that the inhibitory activity is smaller than 1 kDa and the hemozoin pigment has a much higher molecular weight. No inhibitory activity on DCs of the fractions higher than 30 kDa was found. Additionally, it was also shown that the inhibitory activity of the conditioned medium is not sensitive to DNase I treatment, excluding parasite DNA as a mediator of the DCs inhibitory activity.

DCs possess the capacity to secrete immune regulatory cytokines, such as IL-12, which is an important cytokine involved in the initiation of cellular immune responses [58]. It was observed that a soluble factor(s) of *P. yoelii*-infected erythrocytes inhibits the LPS-induced production of IL-12 by DCs. It is not clear yet whether the same parasite-derived factor(s) is responsible for the inhibition of maturation and IL-12 secretion or these two effects are caused by different factors. Regardless, they both probably contribute to impaired T cell responses in the host, since the activation of Th1 cells requires both up-regulation of co-stimulatory molecules on DCs and secretion of IL-12 [58].

Despite parasite inhibition of DC phenotypic maturation, DCs still produce TNF, IL-6 and PGE_2 _in response to *Plasmodium *infected erythrocytes [18,19,38,45]. The *P. yoelii *factor that induces TNF, and at least part of the PGE_2 _and IL-6, was shown to be *Plasmodium*-derived hypoxanthine that is converted into uric acid [38]. Since TNF can induce the maturation of DCs and it was recently shown that systemic TNF in *Plasmodium*-infected mice can impair the DC function [25], it was tested whether hypoxanthine in the conditioned medium or uric acid produced after its degradation were responsible for the impaired maturation of DCs observed.

It was confirmed that *Plasmodium*-derived hypoxanthine degradation into uric acid is not responsible for the inhibition of maturation observed in this study. However, it is remarkable that *Plasmodium *appears to use another small, non-hydrophobic molecule(s) to modulate host DC responses.

Additionally, the *P. yoelii *conditioned medium induces PGE_2 _and IL-6 secretion from DCs, which are important immune mediators during *Plasmodium *infection. PGE_2 _produced during blood stage *P. yoelii *infection has a general T cell inhibitory activity and in particular inhibits protective CD8^+ ^T cell responses against the liver stage [45]. While a specific role of DC IL-6 production during malaria is unknown, IL-6 is considered an important regulator of the transition from the innate to acquired immune responses and it may also influence DC maturation via the inhibition of nuclear factor kappa B (NFκB) [59]. It was shown that *P. yoelii *triggers the early secretion of PGE_2 _in DCs, which in turn induces the secretion of IL-6 later in these cells [19]. Secretion of IL-6 by DCs is in part caused by parasite-derived uric acid, but is in part independent of this pathway [38]. Therefore, the same parasite-derived factor(s) that is responsible for the inhibition of maturation could also contribute to uric acid independent IL-6 secretion.

DCs play a pivotal role in the immune response to pathogens. Using the human malaria parasite *P. falciparum*, human DCs were observed to be modulated [16,18,34]. These findings were supported by field studies in children that showed a decrease in HLA-DR expression on peripheral DCs from children with acute *P. falciparum *malaria [35,36]. Furthermore, it was shown that severe *P. falciparum *infection increases the frequency of BDCA3+ myeloid DCs in the peripheral circulation was increased and the expression of HLA-DR was decreased when comparing children with acute malaria to healthy controls [36].

The findings presented in this study are consistent with those observed in the children with acute *P. falciparum *infection [35,36], with those observed using a human *in vitro *model [16,18,34], and with those observed for murine late infections [17,19,20,42,45,46]. This study indicates that *P. yoelii*-infected erythrocytes inhibit the maturation of DCs at high densities and produce a soluble factor(s) responsible for this effect. During *Plasmodium *infection DCs receive stimulatory signals provided by parasite-derived molecules such as GPI [60], hemozoin-associated parasite DNA [57], elevated concentrations of *Plasmodium*-derived uric acid [38] and probably other molecules that have not been yet characterized. At the same time the parasite appears to release a soluble factor(s) that interferes with the maturation of DCs. It is possible that the balance between these effects is switched during the progression of infection, such that the inhibitory effect may be more active during late infection when parasite loads are higher.

## Conclusion

It was observed that *P. yoelii *infection interferes with the maturation of DCs both *in vivo *and *in vitro*. During late infection, *P. yoelii *inhibits the maturation of murine splenic DCs. *In vitro, P. yoelii*-infected erythrocytes inhibit the maturation of DCs in a dose-dependent manner and also modify their cytokine secretion pattern. A small, heat-stable, non-hydrophobic molecule derived from *P. yoelii*-infected erythrocytes is responsible for the inhibition of DC maturation.

## Authors' contributions

JO, KW, CO and AR conceived the study, participated in its design and coordination and wrote the manuscript. All authors read and approved the final manuscript.
